# Supplementary material for: Characteristics and Comparative Analysis of the Special-Structure (Non-Single-Circle) Mitochondrial Genome of Capsicum pubescens Ruiz & Pav
Source: Genes (Basel). 2024 Jan 24;15(2):152. doi: 10.3390/genes15020152 (PMC10888363; doi:10.3390/genes15020152)
Supplement: Supplementary file 1 [file genes-15-00152-s001.zip › Supplemental figure.pdf]

Materials and Methods

The study selected genes that were present in each sample (namely: atp6, atp9, cob, cox1, mttB, nad3, nad4, nad6, nad7, nad9, rps3, rps4) and concatenated them to construct the phylogenetic tree. Alignment was performed using mafft (v7.427), and the ML tree was constructed using RAxML v8.2.12. The nucleotide substitution model used was GTRGAMMA. The bootstrap value was set to 10,000. The outgroup used was Oryza sativa Indica NC_007886.

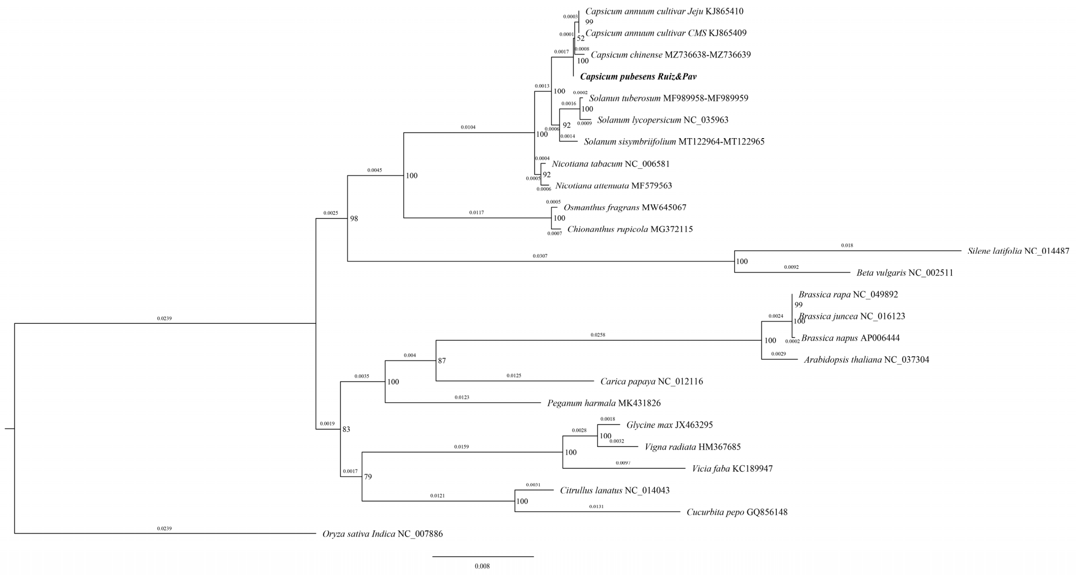

Figure S1 The phylogenetic tree was based on 12 homologous PCGs(atp6 atp9 cob cox1 mttB nad3 nad4 nad6 nad7 nad9 rps3 rps4) in each of the 25 mt genomes.
